# Generalized models for quantifying laterality using functional transcranial Doppler ultrasound

**DOI:** 10.1002/hbm.26138

**Published:** 2022-11-14

**Authors:** Paul A. Thompson, Kate E. Watkins, Zoe V. J. Woodhead, Dorothy V. M. Bishop

**Affiliations:** ^1^ Department of Experimental Psychology Anna Watts Building, Radcliffe Observatory Quarter Oxford UK; ^2^ Present address: Centre for Educational Development, Appraisal and Research (CEDAR) University of Warwick Coventry UK

**Keywords:** fMRI, fTCD, generalized linear model (GLM), generalized additive model (GAM), laterality

## Abstract

We consider how analysis of brain lateralization using functional transcranial Doppler ultrasound (fTCD) data can be brought in line with modern statistical methods typically used in functional magnetic resonance imaging (fMRI). Conventionally, a laterality index is computed in fTCD from the difference between the averages of each hemisphere's signal within a period of interest (POI) over a series of trials. We demonstrate use of generalized linear models (GLMs) and generalized additive models (GAM) to analyze data from individual participants in three published studies (*N* = 154, 73 and 31), and compare this with results from the conventional POI averaging approach, and with laterality assessed using fMRI (*N* = 31). The GLM approach was based on classic fMRI analysis that includes a hemodynamic response function as a predictor; the GAM approach estimated the response function from the data, including a term for time relative to epoch start (simple GAM), plus a categorical index corresponding to individual epochs (complex GAM). Individual estimates of the fTCD laterality index are similar across all methods, but error of measurement is lowest using complex GAM. Reliable identification of cases of bilateral language appears to be more accurate with complex GAM. We also show that the GAM‐based approach can be used to efficiently analyze more complex designs that incorporate interactions between tasks.

## Graphical abstract

Transcranial functional Doppler Ultrasound has been used to assess brain lateralization, but the methods used to extract a laterality index (LI) have involved simple averaging over a period of interest. Here, we compare results from that method with results from a generalized linear models (GLM) approach similar to that used in functional magnetic resonance imaging (fMRI), as well as generalized additive models (GAM) to estimate the cerebral blood flow response. All methods give similar estimates for the mean LI, but error of measurement is much reduced with GAM.

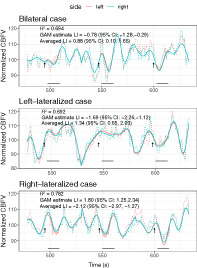

## 1 INTRODUCTION

Lateralization of brain activity (functional lateralization) is usually measured using functional magnetic resonance imaging (fMRI), which provides high spatial resolution, flexible experimental designs, and well‐established analysis procedures. Functional transcranial Doppler sonography (fTCD) is less widely used, but has a number of practical advantages over fMRI, including lower cost, portability, fewer contraindications, and less sensitivity to head motion. While fMRI is often considered the “gold standard” in functional laterality research, the practical advantages of fTCD make it useful for large‐scale studies where the focus is solely on lateralization, regardless of localization of activation within a hemisphere. It is a particularly useful approach for studies that may be challenging using fMRI, including those involving special populations, such as children, or studies involving speech production. Both fMRI and fTCD quantify functional lateralization using a laterality index (LI), but the analytical approach used to calculate LI differs greatly between the two. This article explores how generalized linear models (GLM) and generalized additive models (GAM) may be applied to fTCD data. These methods make it easier to compare results from fMRI and fTCD studies directly, give greater precision of laterality estimates, and increased analytical flexibility over and above what is possible with the current fTCD methods.

We first present a basic description of fTCD and its similarities and differences to fMRI. For more comprehensive details see Badcock and Groen (2017); Deppe et al. (1997); Deppe, Knecht, et al. (2004); Deppe, Ringelstein, and Knecht (2004); Lupetin et al. (1995).

### 1.1 Physiological basis of fTCD versus fMRI

FTCD uses ultrasound probes to detect changes in cerebral blood flow velocity (CBFV) in response to a stimulus. Typically, the stimulus signals that the participant should perform a specific task; for example, presentation of an alphabetic letter requires the participant to mentally generate words that start with that letter. Clinically, fTCD is well‐established as a method for assessing the integrity of the cerebral circulation (Lupetin et al., 1995), but over the past two decades it has been developed as a method for quantifying functional lateralization by researchers (Deppe, Knecht, et al., 2004; Deppe, Ringelstein, and Knecht, 2004; Knecht et al., 1998). The change in CBFV relative to activity in a prior rest baseline period is used as a proxy measure for brain activity. Most commonly, CBFV is recorded from the middle cerebral artery (MCA), which has good coverage of lateral temporal, frontal, and inferior parietal cortical areas (see Figure 2 in Kim et al., 2019), but it is also possible to record from other cerebral arteries. By comparison, fMRI detects the changes in blood oxygenation levels—the blood oxygen level dependent (BOLD) response. The two methods both use indirect measures of brain activity that rely on neurovascular coupling, but whereas fTCD measures perfusion over a widespread arterial territory (Payne, 2017), fMRI detects local changes in the capillaries (i.e., at the spatial scale of individual voxels) (Attwell et al., 2010).

As arterial blood flow and local blood oxygenation are tightly coupled, they should show similar time courses in their response to brain activity (Buxton et al., 1998; Gagnon et al., 2015). The time course of the BOLD response following a brief experimental stimulus has been well characterized (e.g., Boynton et al., 1996; Glover, 1999). It increases to reach a peak at around 5–6 s post‐stimulus, then declines, dropping below baseline levels to reach the trough of the undershoot at around 16 s, before gradually returning to resting levels. This shape can be approximated by a combination of two gamma functions, one modeling the initial peak and one modeling the undershoot (Friston et al., 1998). The precise shape of the response is likely to differ for different individuals and brain regions (Glover, 1999); furthermore, the double gamma function may be insufficient for modeling sustained neural responses to more complex tasks (Frackowiak et al., 2004).

**FIGURE 1:**
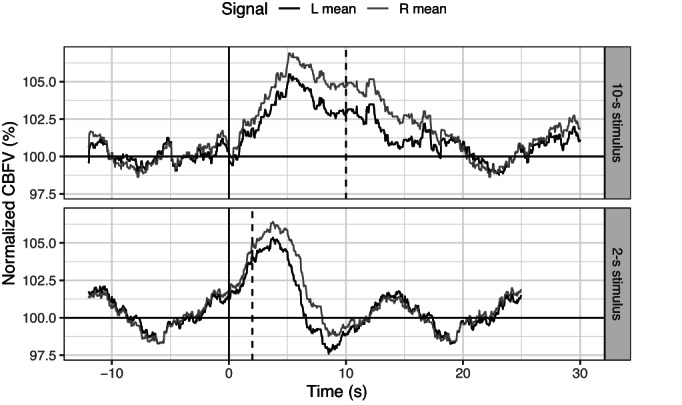
Normalized cerebral blood flow velocity (CBFV) measured with functional transcranial Doppler ultrasound (fTCD) from the left hemisphere (dark grey) and the right hemisphere (light grey) to a 10 s (top) and 2 s (bottom) movement task using the left hand; single illustrative participant averaged over 15 trials. The vertical dashed grey lines indicate the task offset, that is, when the participant finished making the hand movements

The time course of the response in fTCD is not well characterized. Recordings with fTCD of the posterior cerebral artery's CBFV in response to prolonged visual stimulation (Aaslid, 1987; Conrad & Klingelhofer, 1989) showed that (similar to fMRI) the response peaks within four to five. Both studies reported a slow decline in CBFV during the prolonged stimulation. Conrad et al. reported a brief “off‐reaction” (an increase in blood flow) at stimulus offset. For a 20 s stimulus, Aaslid et al. reported that CBFV returned to baseline levels around 6 s after stimulus offset. Conrad et al. reported an undershoot effect after stimulus offset, similar to that seen in fMRI. Figure 1 shows illustrative data that we gathered on a single individual performing a 2‐s hand movement task; this demonstrates a response similar to that seen in fMRI, with an initial peak at ~4 s, and an undershoot which resolved to baseline levels by around 15 s. When a 10‐s stimulus was used, the response continued to build until a peak at around ~5 s, then there was adaptation in the response during the remainder of the task. After task cessation, the signal dropped to baseline by 20 s, followed by an undershoot. Overall, the available evidence suggests that responses observed in fTCD are likely to have a similar shape to those in fMRI, but that the time to peak and to return to baseline may be slightly faster.

### 1.2 Measurement

The main research use of fTCD in healthy individuals has been to assess functional lateralization, that is, differences between the right and left hemispheres. Unlike fMRI, it is not common to focus on activation strength per se, that is, the degree of change in blood flow during task relative to rest. The CBFV recorded in fTCD is measured in meaningful units (cm/s), but the signal amplitude is dependent on both the speed of blood flow and the angle of insonation (the position of the ultrasound probe relative to the artery). Positioning of the probe is subject to operator error (McMahon et al., 2007), and some movement of the probe can occur over the course of an experiment. Hence, there is likely to be a difference in the angle of insonation between the hemispheres, which is adjusted for by a normalization step in the data processing (see below). To avoid unwanted effects of signal drift it is also customary to perform a baseline correction procedure, whereby the data stream is segmented into epochs and mean activation in a period prior to stimulus presentation is subtracted from all values in the epoch. This is comparable to the standard approach used in electroencephalographic studies on event‐related potentials. In contrast, as described below, the modeling approach used in the present article does not involve baseline correction.

### 1.3 Experimental design

FTCD studies typically use experimental designs similar to a block design in fMRI. The focus is on the increase in blood flow in the left and right MCAs associated with a stimulus that signals the start of an experimental task. The length of the block is designed to allow the cerebral blood flow in response to the stimulus to reach a plateau and thus maximize the signal to noise ratio. Sufficient rest time is generally allowed between blocks to let the cerebral blood flow return to prestimulation levels. This limits the number of trials that can be given: an experiment typically consists of around 15–30 trials of a single task, each with the same temporal structure.

Note that the term “baseline” as used in fTCD has a different meaning from that used in fMRI. It merely refers to value of CBFV in the period prior to active stimulation, which is conventionally used to adjust the signal in different epochs to ensure that left and right sides are equalized prior to stimulus presentation. In contrast, in fMRI, the term “baseline” refers to a comparison block in which the participant may either rest or perform a control task that is designed to engage similar perceptual and/or motor processes as the task of interest, allowing brain activation associated with those activities to be removed by subtracting baseline from target activation. Subtraction of different conditions is not typically done with fTCD, where the sole focus is on left–right differences, rather than localization of activity within a hemisphere. In fTCD it is assumed that activation due to factors such as general visual stimulation or motor responses will be removed when the left and right hemisphere signals are subtracted, provided such activation is not itself lateralized. Nevertheless, the key processes that lead to lateralized responses can be hard to identify in fTCD: for instance, if we find a spoken word fluency task gives a lateralized response, we cannot tell how far the mental generation of words and/or their articulation is the key lateralized function. It is necessary to run separate experiments with varying task demands to establish which component processes are lateralized. We show below that with a generalized modeling approach, we can run analyses on fTCD data that are analogous to task comparison as used in fMRI.

As in fMRI, the block design used in fTCD is efficient in terms of signal detection, but has some known issues. First, this design limits which tasks can be used: it is not suitable for tasks that involve infrequent, brief or unpredictable events. Second, performing the same task for the length of a block can allow attention to wander, which is particularly a problem for low‐level perceptual tasks that are not sufficiently engaging. In fMRI, these problems are typically avoided by using an event‐related design, where tasks are presented in individual trials (rather than blocks of trials), with rapid interleaving between different tasks of interest. What this type of design lacks in signal to noise ratio, it makes up for in the number of trials that can be presented in a fixed amount of time (http://imaging.mrc-cbu.cam.ac.uk/imaging/DesignEfficiency). Note, however, it introduces a task‐switching component to the experiment, which may itself generate distinctive neural activation. Event‐related designs are theoretically possible with fTCD, but have not been attempted to date.

### 1.4 Data analysis

The time series data recorded in each trial of fTCD are similar to that of fMRI, but with only one time series per hemisphere (vs. the many voxels per hemisphere in fMRI) and with a higher temporal resolution. FTCD data is acquired at ~100 Hz, although subsequently downsampled (see below), whereas fMRI is acquired at approximately one image every 1–3 s. Data from the two methods are typically analyzed for each participant (within‐subjects) before proceeding to any group comparisons, but the conventional approach is different for fTCD and fMRI.

In conventional fTCD analysis, the data from the two channels are reduced to a single number representing the difference between normalized blood flow velocity for left and right hemispheres. The time series is epoched into individual trials that are time‐locked to stimulus onset; then, after baseline correction, all trials are averaged to reduce the impact of task‐irrelevant noise. A period of interest (POI) is defined, which varies according to task, but typically starts around 3–4 s after the participant is cued to start performing the task and lasts around 15–20 s. The original approach to obtaining a laterality index from fTCD involved identifying the peak value during the POI in the average difference wave formed by subtracting signals from left and right channels (Knecht et al., 1998). Our group found that this generates an artificially bimodal distribution of LIs, and in recent work, we have moved to computing a LI by simply taking the difference between left CBFV and right CBFV, averaged over trials and then temporally averaged over the POI (Woodhead et al., 2018). We refer to this henceforth as the POI averaging method.

In contrast, the usual approach to fMRI analysis uses a general linear modeling (GLM) approach (Koh et al., 2017; Worsley et al., 2002). The time series of blood‐oxygen‐level dependent (BOLD) signal change in each voxel is analyzed separately in a mass univariate analysis. The BOLD signal for the voxel is entered into the GLM as the dependent variable. The predictor is the time course of the onset of trials within the experiment, convolved with the expected shape of the hemodynamic response. This approach predicts how well the time course of the task predicts the time course of signal change in the voxel. The output of the GLM is an estimated beta value for each explanatory variable at that voxel. When a resting baseline is used, the significance of the beta for the task of interest can be converted to a *t*‐statistic to test whether it is significantly greater than zero. Alternatively, when a comparison task (“active baseline”) is used, a contrast can be performed to compare the beta for the task of interest to the beta for the comparison task, again, computing a t‐statistic to indicate the strength (and statistical significance) of the difference.

If the voxel has a *t*‐statistic that exceeds a threshold level, this is taken as a sign of significant voxel activation; a thresholded *t*‐statistic map can be used to highlight specific brain regions that are activated for the task in question. A number of different methods exist for calculating LI from a *t*‐statistic map, but the simplest method is to collect together the subset of *t*‐statistics that exceed the threshold for each hemisphere and sum them to give a relative proportion of activity in each hemisphere (sometimes called “volume of active tissue”). More sophisticated methods have been developed that are not dependent on one critical *t*‐statistic threshold, but instead calculate LI iteratively over a range of *t*‐thresholds and pool across the results; one commonly used method (which we used in the present article) is the bootstrapping method devised by Wilke et al., which is employed in the LI Toolbox (Wilke & Lidzba, 2007; Wilke & Schmithorst, 2006).

### 1.5 Aims

The initial aim of the present article was to evaluate the feasibility of the GLM analysis framework used in fMRI for use with fTCD data from individual participants. Limitations of the GLM approach led us to extend our analysis to include GAMs, which can handle the complex nonlinear time‐series seen with fTCD data. A secondary aim was to demonstrate that these approaches permit a greater range of experimental designs and potential hypotheses to be tested than is currently available. We demonstrate the application of these methods to three existing fCTD datasets using different experimental designs.

In the Methods section, we first describe the three datasets that are used, and the analytic methods for each one. All scripts, data and outputs can be found in the OSF repository (https://osf.io/gw4en/). In the Results section, we compare findings from different model‐fitting approaches with those from the traditional POI averaging method for computing the LI.

## 2 METHODS

### 2.1 Dataset 1

Dataset 1 is from a PhD thesis (Bruckert, 2016), parts of which were published in Bruckert et al. (2021). For the present article, we analyzed data from a word generation task (also known as verbal or phonological fluency), which is widely held as the gold standard measure for assessing language lateralization. A subset of participants were assessed using fMRI: fMRI methods are described in Bruckert (2016) but results from this method have not yet been published elsewhere.

### 2.2 Participants

Data on word generation were available from 154 adult participants (on self‐report: 88 right‐handed females, 45 right‐handed males, 11 left‐handed females, 10 left‐handed males). Mean age was 22 years, range 18–40 years. A subset of 31 participants returned for a fMRI session in which they performed the same word generation task. Those doing fMRI were selected to over‐represent individuals with atypical language laterality, and included 18 left‐handers.

### 2.3 FTCD procedure

Full details of the fTCD procedure can be found in Bruckert et al. (2021). In brief, on each of 23 trials, the participant was shown a letter of the alphabet and asked to silently generate words beginning with that letter for a 5 s period. A second cue at 20 s prompted them to verbally report the words. The POI was specified as 3–20 s after the signal to generate words (allowing for a delay in the blood flow's response to the task).

### 2.4 FMRI procedure

Data were collected using a 3 T Siemens Trio scanner with a 32‐channel headcoil at the Oxford Centre for Clinical Magnetic Resonance Research, University of Oxford. A high resolution T1‐weighted MPRAGE was acquired for image registration (TR = 2040 ms, TE = 4.7 ms, flip angle = 8°, 192 transverse slices, 1 mm isotropic voxels). Echo‐planar images were acquired to measure change in blood oxygen levels during behavioral tasks (TR = 3 s, TE = 30 ms, flip angle = 90°, 48 axial slices, slice thickness = 3 mm, in‐plane resolution = 3 × 3 mm).

Task stimuli were presented using Presentation Software (Neurobehavioral Systems) with stimulus onset synchronized with the scanner. The stimuli were projected via a mirror mounted on the head coil. The task was performed similarly to in fTCD, but due to fMRI's greater susceptibility to motion artifacts there was no overt word reporting phase. A block design was used, where the task was performed for 15 s followed by 15 s of rest (with a fixation cross). There were 12 blocks, each with a different letter presented on the screen throughout the duration of the block. Participants were required to covertly think of as many words as they could starting with that letter.

### 2.5 FMRI analysis

Analysis of fMRI data was conducted using FEAT (the fMRI Expert Analysis Tool) in FSL (FMRIB Software Library, http://www.fmrib.ox.ac.uk/fsl). The pre‐processing stages included head motion correction through realignment to the middle volume of the EPI dataset; skull stripping using FSL's Brain Extraction Tool (Smith, 2002); spatial smoothing using a 6 mm full‐width‐half‐maximum Gaussian kernel; high‐pass temporal filtering at 90 s; and unwarping using fieldmaps in FSL's Phase Region Expanding Labeller for Unwrapping Discrete Estimates tool (PRELUDE) and FMRIB's Utility for Geometrically Unwarping EPI (FUGUE; Jenkinson, 2003).

The pre‐processed data were entered into first‐level (subject‐specific) GLMs. The explanatory variables (EVs) in the GLM were: the timings of the word generation blocks convolved with a double‐gamma function; the temporal derivatives of the time course EV; and six motion correction parameters as covariates of no interest. The contrast of interest was word generation versus the implicit (resting) baseline.

The z‐statistic maps from the contrasts were used to calculate LI values using the bootstrapping method in the LI Toolbox (Wilke & Lidzba, 2007; Wilke & Schmithorst, 2006). The LI values were calculated for a combined mask of the frontal, temporal, and parietal lobes as an approximation of the MCA territory. The mask excluded 5 mm either side of the midline and was created using templates provided in the LI Toolbox. Although it is more conventional to use *t*‐statistics, when the degrees of freedom exceed 30, z‐statistics are numerically very similar, but benefit from having a more normal distribution.

LIs for fMRI are conventionally calculated using the formula, LI = (L − R)/(L + R). This traditional LI has one limitation, namely that it is bounded by −1 and 1, whereas the LI from fTCD is unbounded. The correlation with LIs from fTCD is therefore likely to be deflated by restriction of range. To obtain a measure more analogous to the fTCD LI, we computed a difference score (L − R) based on the number of suprathreshold voxels within the masked region in the left and right hemispheres.

### 2.6 FTCD data pre‐processing

We first applied the generic processing steps used in our original studies, which are similar to the pipeline proposed by Deppe et al. (1997). Raw data were downsampled from 100 to 25 Hz: this value is arbitrary, based on prior work showing that this level of precision is more than adequate to capture task‐related changes in the signal. The data were then epoched in relation to marker signals that correspond to the stimulus presentation. The left and right signals were normalized to a mean of 100 by dividing by the respective channel mean. Heart cycle integration was performed by identifying regular peaks in the waveform and averaging over the peak‐to‐peak interval. Extreme values (normalized scores below 60 and above 140) were discarded. Baseline correction was performed for each epoch separately for data from the left and right hemispheres by subtracting the mean value during the baseline period from the signal across the whole epoch, and the averaged LI was computed as the mean of the difference wave over the POI (8–20 s from start of the epoch). A *SE* for the averaged LI was obtained by computing LI for individual trials and taking the *SE* of the estimates.

**FIGURE 2:**
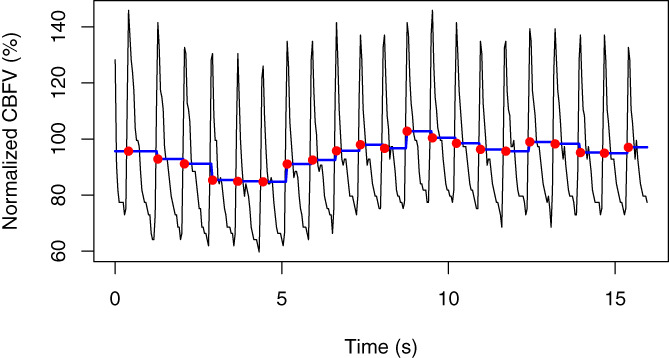
Portion of data analyzed for Dataset 1 (see below). Raw signal (black lines) showing peaks in cerebral blood flow velocity (CBFV) with each heartbeat, and signal after heart cycle integration (blue lines). Red dots show one point per heart cycle, as used in our analysis

After heart cycle integration, the resulting data have a block‐stepped appearance with high autocorrelation (see Figure 2). This is not ideal for the GLM approach, so we ameliorated this by further downsampling so that each heartbeat contributed one value. The effective sampling rate depends on the individual's heart rate, but is typically between 1 and 1.5 Hz. Note that for the model‐fitting approach, we did not use baseline corrected data.

### 2.7 GLM analysis of fTCD data

The classic GLM analysis for a block design in fMRI fits the following model to the observed time series (*y*) to estimate values of *β* at each voxel:

(1) $$y=\beta_{0}+\beta_{1}.x+\varepsilon$$

where *y* is observed activation, *x* is the predicted hemodynamic response function, and *ε* is error.

Because we have only two series of observations, from left and right respectively, a simple way to obtain a laterality index with fTCD is to modify this model as follows:

(2) $$y=\beta_{0}+\beta_{1}.x+\beta_{2.}\text{hemisphere}+\beta_{3}.x^{*}\text{hemisphere}+\varepsilon$$

where hemisphere is coded as 1 for the CVFV from the left or − 1 for right. The coefficient *β*_3_ is then a direct estimate of the laterality index.

We modeled the hemodynamic response (HDR) function, *x*, using the *fmri* package in R (Tabelow & Polzehl, 2011) to estimate a gamma function with default settings. We estimated two HDR functions; one starting at the stimulus for word generation, and a second one corresponding to the signal to report the generated words. Studies using word generation with fTCD have found that it is the word generating phase, rather than the reporting phase, that gives strong left‐lateralization in most people, and accordingly, the laterality index was estimated as coefficient for the interaction term (*β*_4_) in the following equation:

(3) $$\begin{array}{l} y=\beta_{0}+\beta_{1}.\text{HDR1}+\beta_{2}.\text{HDR2}+\beta_{3.}\text{hemisphere}+\beta_{4}.\\ \mathrm{HDR}1^{*}\text{hemisphere}+\beta_{5}.t+\beta_{6}.t^{2}+\beta_{7}.\\ t^{3}+\varepsilon \end{array}$$

where *t* is time in seconds from the start of the experiment. Consistent with use of GLM in fMRI analysis (Worsley et al., 2002), to allow for signal drift across the session, we included terms for linear, quadratic, and cubic effects of time (*t*).

### 2.8 GAM analysis of fTCD data

Rather than estimating the HDR, we could directly estimate it from the data, by switching to a GAM that allows us to model nonlinear trends in the data by incorporating splines to model the time components (one model per individual). We therefore considered whether we might obtain more precise estimates of the laterality index by adding additional predictors, moving to a GAM approach. With a GAM, we still need to specify a time period during which a lateralized response is predicted, and we used the same POI as specified using the POI Averaging method (i.e., 3–20 s after the cue to generate words).

A useful introduction to GAMs can be found in Pedersen et al. (2019). As with GLM, the response, *y*, is predicted by a linear combination of explanatory variables, but GAM combines both parametric and nonparametric terms. This gives a hybrid or semiparametric model which incorporates nonlinear trends in the time predictors, while maintaining parametric terms for predictors, such as hemisphere and stimuli by hemisphere interactions. The flexible nonlinear fit is achieved using a smoother function, $f\left(x\right)$ (Green & Silverman, 1993; Hastie & Tibshirani, 1986), which consists of a set of smaller functions which are referred to as basis functions. The smoother function, *f*(*t*), can be written as the sum of K simpler, fixed basis functions ($b_{i,k}$) with corresponding coefficients ($\beta_{i,k}$), as follows:

$$f_{1,i}\left(t_{i}\right)=S\sum_{k=1}^{K}\beta_{i,k}b_{i,k}\left(t_{i}\right)$$

*K* refers to the number of knots in a spline, which determines the complexity or “wiggliness” of the smoother. With this increased flexibility, there is a risk of overfitting, so a smoothing penalty is introduced to control for this. Each smoother has its own penalty matrices (S) according to the basis functions included, which are multiplied by the vector of estimated β coefficients to form the penalty, $\beta^{T}\mathrm{S\beta}$. The GAM model is fitted using penalized estimation, specifically the penalized maximum likelihood:

$$l_{p}\left(\beta \right)=l\left(\beta \right)-\text{penalty}=l\left(\beta \right)-\lambda \beta^{T}\mathrm{S\beta}$$

A smoothing parameter (λ) is also introduced to control the influence of the smoothing penalty. In our GAM model, we used the default thin‐plate splines from the R package *mgcv* (Wood, 2017) as the smoother in the model. The benefit of this type of spline is that the position of the knots is determined from the data. This avoids the need to prespecify the number of knots and their location prior to model fitting. Further details of the smoother and penalized likelihood specifications can be found in Green and Silverman (1993) and Wood (2003, 2017).

To apply this kind of model, we first divided the data into epochs, and then incorporated smoothers both for total time elapsed from the start of the session (*t*), and relative time within the epoch (*r*). The laterality index is estimated from the interaction between hemisphere and the boxcar function that indicates the time period when the lateral difference is expected, that is, the original POI. The simpler version of this model is shown in Equation (4):

(4) $$y=\beta_{0}+\beta_{1}.s\left(t\right)+\beta_{2}.s\left(r\right)+\beta_{3}.\mathrm{POI}+\beta_{4.}\text{hemisphere}+\beta_{5}.{\mathrm{POI}}^{*}\text{hemisphere}+\varepsilon$$

where *t* is time from the start of the experiment, *r* is time relative to the start of the epoch, POI is the period of interest for this task (coded 1 or 0), and s() denotes a smoother. We refer to this model as simple GAM.

The model from Equation (4) is sensitive to regularities in the signal outside the POI, provided they are consistent across epochs. A more complex version of the model was also run, where epoch, coded as a factor that interacts with relative time, was included as a predictor, substituting for absolute time (*t*), so epoch‐specific variation was modeled. This model, which we refer to as the complex GAM, is:

(5) $$\begin{array}{l} y=\beta_{0}+\beta_{1}.s\left(r\right)+\beta_{2}.s\left(r,\text{epoch}\right)+\beta_{3}.\mathrm{POI}+\beta_{4.}\text{hemisphere}+\beta_{5}.\\ {\mathrm{POI}}^{*}\text{hemisphere}+\varepsilon \end{array}$$

The rationale for model (5) is that cerebral blood flow can be influenced by behaviors that may be inconsistent across epochs; for instance, activities such as drawing breath, which is not lateralized, but has a large impact on the signal. By modeling such features of the data, rather than subsuming them in the noise term, we may obtain a more precise estimate of the LI. However, we need to be cautious in extending the model this way, as there is a risk of overfitting. As discussed above, the GAM method is designed to counteract over‐fitting, and in practice the results of the model‐fitting can also be used to evaluate the suitability of the model to capture the phenomena of interest. In particular, we can see how reliable the LI estimates are across repeated measurements for the same individual: if we are just overfitting random noise, then reliability should be poor.

In both GAM models, the laterality index is derived from the magnitude of the interaction between hemisphere and POI, which gives an estimate of the difference in response between the two sides.

### 2.9 Dataset 2

Woodhead et al. (2021) reported fTCD data on a set of language activation tasks for participants given the same test battery on two occasions. Here, we analyzed data from a sentence generation task, based on Mazoyer et al. (2014), which was the most lateralized task in the battery.

#### 2.9.1 Participants

Data were available for 73 adults aged between 18 and 45 years (30 left‐ and 43 right‐handers) who were tested on two occasions to establish test–retest reliability of language laterality. The interval between sessions ranged from 3 days to 6 weeks.

#### 2.9.2 FTCD procedure

FTCD data were acquired using the same apparatus as Bruckert (2016). The task followed a similar procedure to the word generation task described above. On each of 15 trials, participants saw “CLEAR MIND” for 3 s; followed by a black and white line drawing for 3 s; then a fixation cross for 11 s; a “REPORT” prompt for 6 s; and finally, a “REST” prompt for 10 s. Participants were required to covertly (silently) generate a sentence to describe the picture, then say it overtly (aloud) when they saw “REPORT.” The data were analyzed using the same script as for the Bruckert sample, with a baseline period from 5 s prior to the “CLEAR MIND” cue, to 2 s after it. The POI was from 6 to 17 s after the “CLEAR MIND” signal (corresponding to the covert sentence generation part of the task). Data and materials can be found on OSF: https://osf.io/tkpm2/.

#### 2.9.3 FTCD data pre‐processing

The same methods were used as for Dataset 1.

#### 2.9.4 FTCD GLM and GAM analysis

The same methods were used as for Dataset 1.

### 2.10 Dataset 3

Woodhead et al. (2018) used three different language tasks within one fTCD session. This study was influenced by Mazoyer et al. (2014), who conducted an fMRI study of language laterality subtypes. They computed a laterality index where list generation was used as a comparison (baseline task) for sentence generation. In effect, the aim was to obtain a measure of lateralization for generation of sentences that controlled for the articulatory processes involved in producing single, overlearned words. Woodhead et al. (2018) used analogous tasks, making it possible to explore the application of the GAM method to quantifying the interaction between laterality and task with fTCD data.

#### 2.10.1 Participants

Participants were 31 adults (29 right‐handers; 20 females; age mean = 25 years).

#### 2.10.2 FTCD procedure

Within one test session, participants were given three tasks: word generation (WG), sentence generation (SG), and list generation (LG), with task blocks presented in a predetermined pseudorandomized order within each participant. The session was broken into two runs, with 10 trials of each task in each run. The three tasks (WG, SG, and LG) followed a common temporal structure, as described below. The WG and SG tasks were as described above. In LG, participants saw a scrambled line drawing image, and were required to covertly (silently) rehearse the numbers from one to 10, then say them aloud when they saw the “REPORT” cue. Data and materials can be found on OSF: https://osf.io/pq6wu/.

#### 2.10.3 FTCD data pre‐processing

The fTCD data were pre‐processed as for Datasets 1 and 2. The baseline period was the 5 s of rest prior to the “CLEAR MIND” cue. The POI was from 7 to 17 s after the CLEAR MIND cue.

#### 2.10.4 FTCD GAM analysis

Our focus was on the contrast of the laterality indices for sentence generation and list generation. First, following Woodhead et al. (2018), we estimated LIs (and corresponding *SE*s) for the list generation and sentence generation tasks using the POI averaging method. The LIs for the two tasks were compared using a *t*‐test.

Next, we modified the complex GAM approach by a simple extension of the regression formula to include a term that represents the three‐way interaction between POI, task and hemisphere, and associated two‐way interactions, that is:

(6) $$y=\beta 0+\beta_{1}.s\left(r\right)+\beta_{2}.s\left(r,\text{epoch}\right)+\beta_{3}.\mathrm{POI}+\beta_{4}.\text{hemisphere}+\beta_{5}.\text{task}+\beta_{6}.{\mathrm{POI}}^{*}\text{hemisphere}+\beta_{7}.{\mathrm{POI}}^{*}\text{task}+\beta_{8}.{\text{hemisphere}}^{*}\text{task}+\beta_{9}.{\mathrm{POI}}^{*}{\text{hemisphere}}^{*}\text{task}+\varepsilon$$

In our updated analysis (model 6), we specified LG as the first level of the task factor, against which other tasks were compared, giving an interaction term between the LI estimate (two‐way interaction POI and hemisphere) and task that reflected the difference between SG and LG LIs.

### 2.11 General criteria for evaluating models

There is no single gold standard for evaluating adequacy of a brain lateralization measure. We present data on the following criteria:

- Left‐lateralization of language activation at the population level. As our focus is on language tasks, we expect the distribution of LIs to show a bias to the left. Note that the *SE* of LI *within* the sample is not of particular interest, as this will depend on the proportion of people who are atypically lateralized.
- Error of measurement of LI *within* individuals. The smaller the *SE* of the within‐individual LI, the more confident we can be that it is an accurate representation of a person's lateral bias. In many studies, the LI is converted into a categorical measure, with a division between left‐lateralized, right‐lateralized, or bilateral individuals. This can be done by considering whether the confidence interval around the LI crosses zero. When the *SE* is high, it is difficult to distinguish genuine bilaterality from lateral bias with noisy measurement. The proportion of individuals categorized as having bilateral language may be taken as an index of precision of measurement, where a high proportion corresponds to an imprecise measure.
- Proportion of variance accounted for. For GLM and GAM models, we can consider R2 as a measure of proportion of variance accounted for by the model. Note, however, that this inevitably rises as additional parameters are included in a model; hence, it is not a suitable measure for comparing models, although it does give an indication of absolute level of fit.
- Akaike Information Criterion (AIC). This is a measure of goodness of fit of a model, adjusted for the number of predictors in the model, which can be used to compare models. The best‐fitting model is the one with the lowest AIC, that is, the one that explains the greatest amount of variation using the fewest possible independent variables.
- Bayesian Information Criterion (BIC). This is similar to the AIC, in that it estimates fit of a model while taking into account the number of predictors, but it uses Bayesian rather than frequentist probability. Again, the lower the BIC, the better the fit of the model.
- Agreement with laterality as assessed using fMRI. For Dataset 1, a subset of individuals had done the same task with both fTCD and fMRI, providing an opportunity to compare the LI estimates from the two methods.
- Test–retest reliability of LI. In Dataset 2, the same individuals had repeated fTCD language assessment on two occasions, so we could estimate test–retest reliability of LI as assessed using different methods. In addition, we considered agreement between categorical laterality (left, right, and bilateral) across two sessions, using the confidence interval to assign laterality as described in (b) above.
- For Dataset 3, we compared the sensitivity of the GAM approach (model 6) to a simple subtraction of LIs for the two tasks computed using the POI Averaging method.

## 3 RESULTS

### 3.1 Dataset 1

**FIGURE 3:**
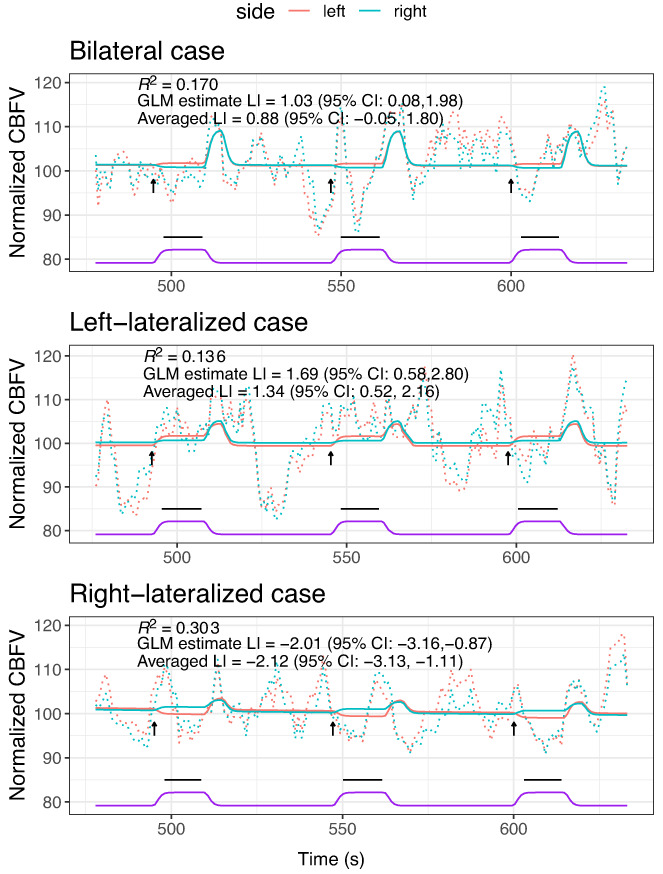
Sample data from three epochs for three illustrative cases. The predicted cerebral blood flow velocity (CBFV) from the generalized linear model (GLM) is shown as solid blue (right) and pink (left) lines; observed values are shown as dotted lines. The arrows show the time where the task started, and the conventional period of interest (POI) is shown as black horizontal lines below. The purple trace is the hemodynamic response (HDR) function (arbitrary units). Averaged laterality index (LI) is the LI from the POI averaging method, and GLM estimate LI is value from the interaction term in the GLM. R2 is proportion of variance accounted for by the model. These estimates of LI and R2 are based on analysis of all 23 epochs

For Dataset 1, an arbitrary series of three epochs in the middle of the series (epochs 10–12) was selected to visualize the fit of the GLM model‐fitting function in individual participants: three participants were selected as exemplars of individuals who had clear left‐lateralization, clear right‐lateralization, and bilateral language, respectively, on the POI averaging approach. As can be seen from Figure 3, the fit of the predicted function is generally unimpressive, and the percentage of variance accounted for by the model is poor, although the LI estimates from the GLM appear similar to those from the original POI averaging method. Nevertheless, note that the first case, who was categorized as having bilateral language by the POI averaging method (i.e., the confidence interval spanned zero) is significantly left‐lateralized when the GLM approach is used. In Table 1, we show more detailed comparisons of the performance of the GLM versus the conventional approach.

**TABLE 1:** Mean (95% CI) for indicators of model fit for Dataset 1 (*N* = 154)

Index	POI average	GLM	GAM	GAM2
Mean LI	2.15 [1.95, 2.35]	2.10 [1.92, 2.27]	1.94 [1.78, 2.10]	1.94 [1.78, 2.10]
Within‐subject *SE* LI	0.52 [0.50, 0.54]	0.56 [0.55, 0.58]	0.53 [0.52, 0.55]	0.37 [0.36, 0.38]
*R* ^2^	‐	0.20 [0.19, 0.22]	0.30 [0.29, 0.32]	0.65 [0.64, 0.66]
AIC/1000^a^	‐	18.5 [18.1, 19.0]	18.1 [17.7, 18.6]	16.3 [15.9, 16.8]
BIC/1000^a^	‐	18.6 [18.2, 19.0]	18.3 [17.9, 18.7]	17.5[17.1, 18.0]
% Right	5.2	1.9	1.9	3.2
% Bilateral	11.7	15.6	14.3	8.4
% Left	83.1	82.5	83.8	88.3
*r* with POI average	‐	0.93 [0.91, 0.95]	0.92 [0.89, 0.94]	0.92 [0.89, 0.94]
*r* with fMRI LI (*N* = 31)	0.61 [0.33, 0.80]	0.69 [0.45, 0.84]	0.67 [0.41, 0.83]	0.67 [0.41, 0.83]
*r* with fMRI L‐R	0.71 [0.47, 0.85]	0.72 [0.49, 0.85]	0.71 [0.48, 0.85]	0.71 [0.48, 0.85]

Abbreviations: AIC, Akaike Information Criterion; BIC, Bayesian Information Criterion; CI, confidence interval; fMRI, functional magnetic resonance imaging; LI, laterality index; GAM, generalized additive model; GLM, generalized linear model; POI, period of interest.

^a^ divided by 1000 to facilitate formatting.

Table 1 compares the LI obtained from the GLM interaction from model (3) with that obtained using the original POI Averaging method. The means are closely similar, but the *SE* of the LI estimate is somewhat higher with the GLM method than with the original method. As noted above, the 95% confidence interval around the LI can be used to identify those with bilateral language (i.e., people whose confidence interval spans zero), and the higher *SE* means that the GLM method would place more people in this category than the original averaging method.

**FIGURE 4:**
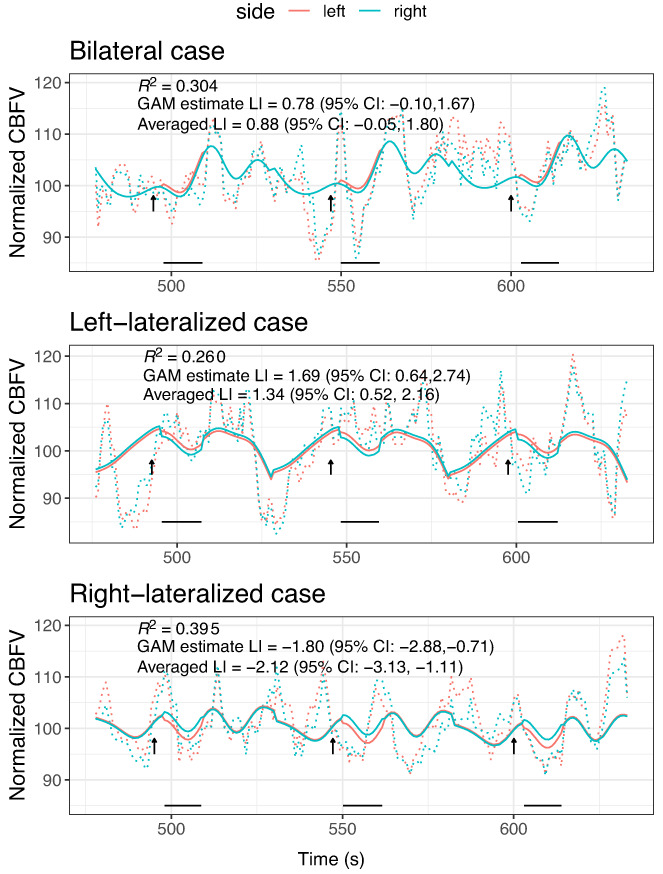
Sample data from three epochs for same three illustrative cases as Figure 3. The predicted cerebral blood flow velocity (CBFV) from the generalized additive model (GAM) excluding epoch (Equation (4)) is shown as solid blue (right) and pink (left) lines; observed values are shown as dotted lines. The arrows show the time where the task started, and the period of interest (POI) is shown as black horizontal lines below. Averaged laterality index (LI) is the LI from the conventional averaging over the POI, and GAM estimate LI is value from the interaction term in the GAM. R2 is proportion of variance accounted for by the model. These estimates of LI and R2 are based on analysis of all 23 epochs

**FIGURE 5:**
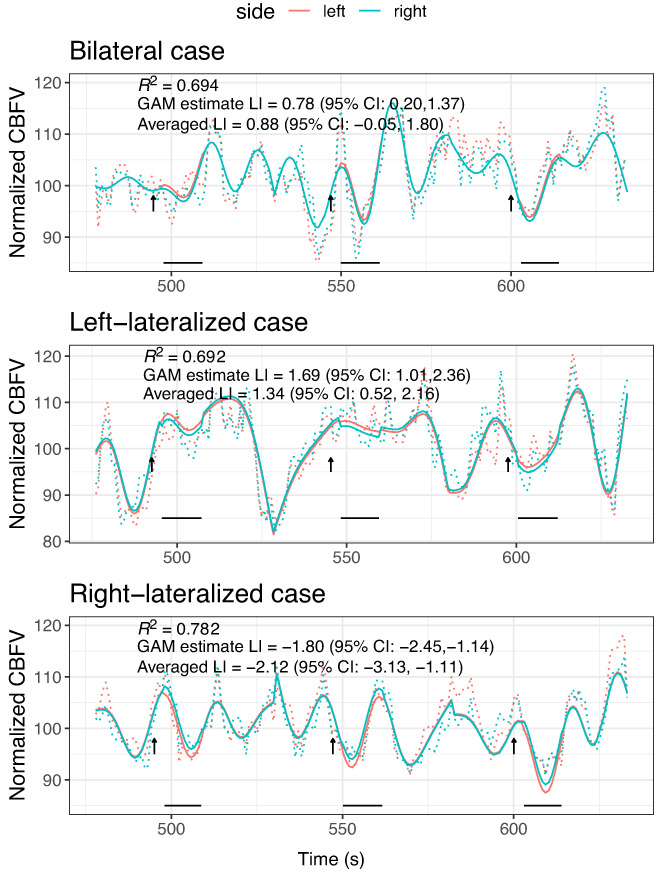
Sample data from three epochs for same three illustrative cases as Figure 3. The predicted cerebral blood flow velocity (CBFV) from the generalized additive model (GAM) including epoch (Equation (5)) is shown as solid blue (right) and pink (left) lines; observed values are shown as dotted lines. The arrows show the time where the task started, and the POI is shown as black horizontal lines below. Averaged laterality index (LI) is the LI from POI averaging, and GAM estimate LI is value from the interaction term in the GAM. R2 is proportion of variance accounted for by the model. These estimates of LI and R2 are based on analysis of all 23 epochs

Illustrative fits of the two GAM models for the same 3 epochs as Figure 3 are shown in Figures 4 and 5, indicating much improved prediction, as would be expected from this kind of model. The simple GAM (Equation (4)) captures regularities across the time frame of the epoch; the more complex GAM (Equation (5)) also incorporates idiosyncratic variation for each epoch and so achieves much closer fit.

Table 1 shows the quantitative fit statistics for these models. It is apparent from the AIC and BIC values that model fit is superior for the more complex GAM model that includes epoch as a predictor, even after taking into account the larger number of predictors in the model. The estimates of LI are not very different from the simple POI averaging method, but the SEs of these estimates decline going from the GLM to the simple GAM to the complex GAM. It follows that the latter model also categorizes the smallest number of cases as having bilateral language, suggesting that the other models may be miscategorizing those with noisy data as bilateral.

#### 3.1.1 Comparison with fMRI

**FIGURE 6:**
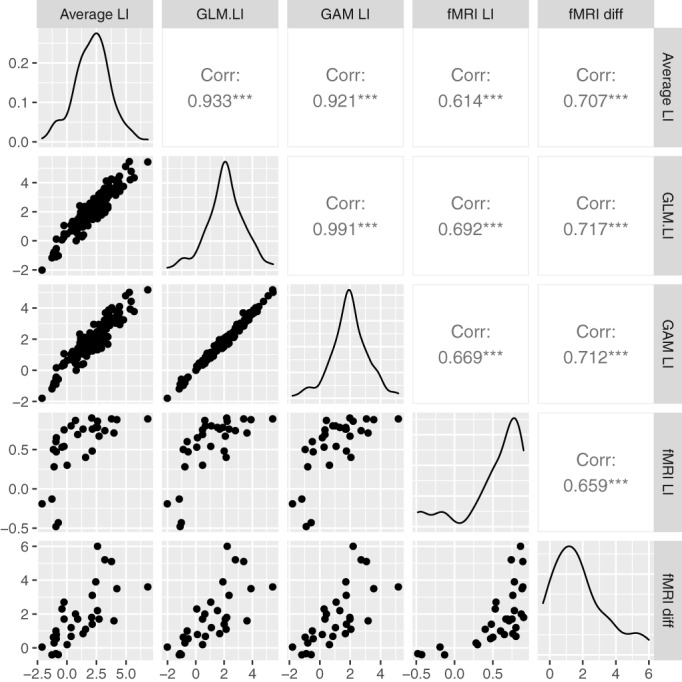
Scatterplots and density plots comparing the period of interest (POI) average, the three methods of computing laterality index (LI) from functional transcranial Doppler ultrasound (fTCD) data (Dataset 1) and the LI and difference score from functional magnetic resonance imaging (fMRI) for 31 participants. The generalized additive model (GAM) LI is based on the complex model including epoch. The fMRI difference score is divided by 10,000. The density plots on the diagonal show the distribution of the measure in the right‐hand label.

Because Bruckert (2016) gave the same task to a subset of participants using fMRI, we were able to compare the model‐fitting results with those from standard GLM analysis of fMRI data on the same task, using the same hemodynamic response function. Although the correlation is numerically slightly higher for the GLM than the averaged method, the estimates are not reliably different: both show moderate agreement between methods. Scatterplots for the LIs from the average method, GLM and complex GAM and the fMRI LI and difference measure are shown in Figure 6. (The LIs from the simple GAM are so similar to those from complex GAM that they are not plotted).

### 3.2 Dataset 2

Table 2 shows results for Dataset 2 in a format similar to Table 1 for the different methods. Note there is no fMRI data for this sample.

**TABLE 2:** Mean (95% CI) for indicators of model fit for Dataset 2 (*N* = 73 participants × 2 sessions)

Index	POI average	GLM	GAM	GAM2
Mean LI	2.37 [2.08, 2.66]	2.13 [1.88, 2.38]	1.90 [1.67, 2.14]	1.90 [1.67, 2.14]
Within‐subject SE LI	0.66 [0.63, 0.69]	0.76 [0.74, 0.78]	0.67 [0.65, 0.69]	0.38 [0.37, 0.39]
*R* ^2^	‐	0.14 [0.13, 0.15]	0.30 [0.28, 0.31]	0.76 [0.75, 0.77]
AIC	‐	7784 [7579, 7989]	7540 [7342, 7738]	6285 [6119, 6452]
BIC	‐	7830 [7624, 8035]	7651 [7453, 7850]	6965 [6793, 7137]
*r* with POI Average	‐	0.97 [0.96, 0.98]	0.95 [0.93, 0.97]	0.95 [0.93, 0.97]
Test–retest r	0.82 [0.73, 0.88]	0.81 [0.71, 0.88]	0.81 [0.72, 0.88]	0.81 [0.72, 0.88]
% Inconsistent	6.8	12.3	11.0	8.2
% Consistent right	8.2	4.1	5.5	8.2
% Consistent bilateral	8.2	12.3	9.6	2.7
% Consistent left	76.7	71.2	74.	80.8

Abbreviations: AIC, Akaike Information Criterion; BIC, Bayesian Information Criterion; CI, confidence interval; fMRI, functional magnetic resonance imaging; LI, laterality index; GAM, generalized additive model; GLM, generalized linear model; POI, period of interest.

As with Dataset 1, the estimates of LI are similar for the original averaging method and those obtained by fitting a generalized model, with correlations of .95 or more between methods. Note that the within‐subject *SE* values are higher for the first three methods than for Dataset 1: this reflects the smaller number of trials used in the SG task (*N* = 15) compared with the WG task of Dataset 1 (*N* = 23). With the complex GAM method, the within‐subject *SE* is again much smaller, and comparable to that obtained with Dataset 1.

As we have two measures for each participant, test–retest reliability is shown in the lower section of Table 2. Given the good agreement for LI estimates across methods, it is not surprising that the reliability is also similar across methods, all of which show good consistency of measurement across occasions. Finally, at the bottom of the table, we see laterality categorized as either consistent across sessions or inconsistent. The method is as described above under General criteria for evaluating models, that is, if the 95% confidence interval spanned zero, the case was categorized as bilateral; otherwise the direction of the LI was used to categorize as left or right‐lateralized. The proportions of inconsistent cases are similar across models, but it is noteworthy that the highest level of consistency is seen for the complex GAM model; we would not expect such consistency across sessions if the good fit was merely a consequence of overfitting.

### 3.3 Dataset 3

**FIGURE 7:**
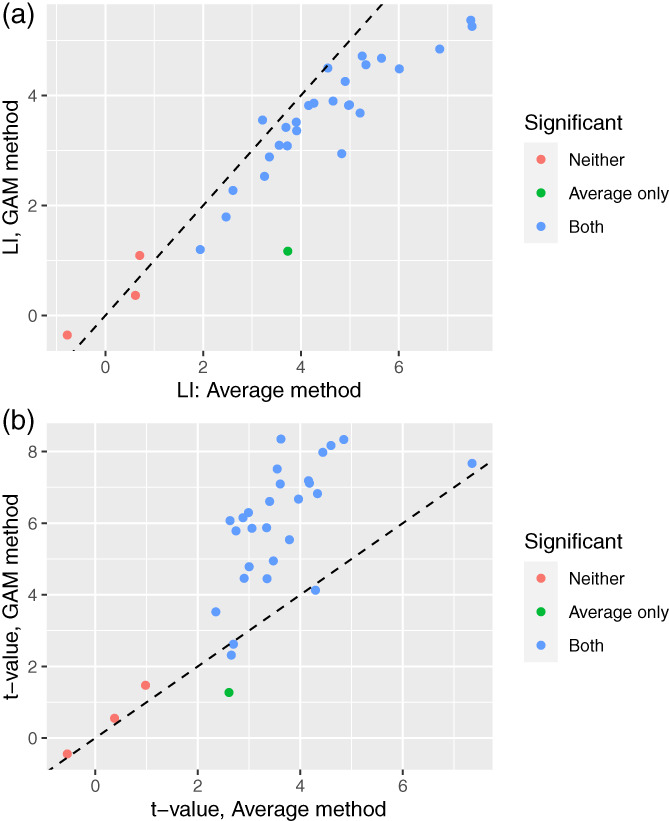
Comparison of results from sentence‐list laterality index (LI) contrast (Dataset 3) for period of interest (POI) average method (x‐axis) and complex generalized additive model (GAM) (y‐axis), with panel A showing LI contrast estimates, and panel B showing *t*‐values. The color‐coding indicates whether the *t*‐test for the contrast gives *p* < .05 for neither method, for the POI average method only, or for both methods

Figure 7a shows the scatterplot for the LI, based on SG corrected for LG, both using the POI averaging method (subtracted means) and the GAM model. Figure 7b shows the corresponding *t*‐values. It is evident from inspection that the two methods give similar results. The POI averaging method gives slightly higher values for LI, but the *t*‐values are higher when the GAM method is used. This again reflects the tighter confidence interval around estimates when the GAM method is used.

## 4 DISCUSSION

In this article, we evaluated new statistical analyses for transcranial Doppler ultrasound data that more closely align with analysis techniques used in fMRI: first a GLM incorporating a hemodynamic response function as predictor, and then two GAMs that estimate the response function from the data.

The first point to note is that LIs estimated using the conventional POI averaging method agree well with those from the more sophisticated model‐fitting methods. In terms of test–retest reliability (Dataset 2) and correlation with fMRI laterality (Dataset 1), the POI averaging method performed as well as model‐based approaches. It follows that previous studies that used individual POI averaging LIs in group analyses perform well when compared to more sophisticated approaches to measurement, which increases confidence in findings from the fTCD literature to date.

On the other hand, it is clear from analyses of all three datasets considered here, that at the individual level, the estimate of LI is more precise when a complex GAM model‐fitting approach is used. The smaller confidence intervals around the LI estimate from the complex GAM imply that when individuals are categorized as having left‐, right‐, or bilateral language, fewer cases will be erroneously classified as bilateral just because their data is noisy. In addition, as shown in the analysis of Dataset 3, at the individual level, the GAM approach is more sensitive at detecting significant differences in laterality between task conditions.

A complex GAM approach that models epoch‐related variation offers considerable potential for obtaining precise individual estimates of LI, as well as providing a simple way of incorporating task contrasts in the analysis.

In principle, this method offers further potential, opening up the possibility of using a range of experimental designs that are commonly used in the context of fMRI. It would, for instance, be straightforward to apply the GAM approach to a design using parametric modulation of a task rather than categorical task differences. In that case, one would simply substitute a quantitative measure for the “task” term in model (6). In addition, the GAM approach lends itself to event‐related designs. Direct comparison of results from fMRI and fTCD studies have the potential to throw light on underlying processes driving cerebral lateralization by combining information on spatial localization and time course of activation. Nonetheless, we may note that extension of the GAM model to fMRI would pose difficulties because the computational load of such analysis becomes substantial when more than two channels of data are involved.

## 5 CONCLUSIONS

The potential benefits of employing a GAM model‐based analysis for fTCD data are: more precise estimates of the laterality index, making it easier to draw a distinction between noisy data and bilaterality; flexible options for study design, for example, event‐related designs or parametric modulators; the possibility of performing contrasts between tasks, for example, to use active rather than resting baselines; biologically plausible modelling of the neurovascular response; and, finally, more direct comparability to fMRI analysis methods.

## AUTHOR CONTRIBUTIONS

**Paul A. Thompson**: Conceptualization, methods development and statistical analysis, writing ‐ original draft preparation, writing ‐ review and editing; **Kate E. Watkins**: conceptualization, writing ‐ review and editing; **Zoe V. J. Woodhead**: Conceptualization, methods development, writing ‐ original draft preparation, writing ‐ review & editing. **Dorothy V. M. Bishop**: Conceptualization, methods development & statistical analysis, writing ‐ review and editing; All figures have been created by the authors.

## FUNDING INFORMATION

This work was supported by a European Research Council Advanced Grant (694189). The funders had no role in study design, data collection and analysis, decision to publish, or preparation of the manuscript.

## CONFLICT OF INTEREST

The authors declare no conflict of interests.

## Data Availability

All R scripts and datasets are available at the Open Science Framework repository: https://osf.io/gw4en/
